# Dissection aortique anévrismale chez un adulte infecté par le VIH-1 dans le cadre d'un syndrome de reconstitution immune avec tuberculose

**DOI:** 10.11604/pamj.2018.31.10.12824

**Published:** 2018-09-04

**Authors:** Desmorys Raoul Moh, Anani Badjé, Nogbou Frederic Ello, Jean-Baptiste N'takpé, Jean-Baptiste Anzouan-Kacou, Gérard Menan Kouamé, Simplice Ackoundzé, Franck Boccara, Olivier Ba-Gomis, Serge-Paul Eholié, Xavier Anglaret, Christine Danel

**Affiliations:** 1 Programme PAC-CI, site ANRS de Côte d'Ivoire, CHU de Treichville, Abidjan, Côte d'Ivoire; 2 Département de Dermatologie et Infectiologie, Université Félix Houphouët-Boigny Abidjan, Côte d'Ivoire; 3 Service de Maladies Infectieuses et Tropicales, CHU de Treichville, Abidjan, Côte d'Ivoire; 4 Université de Bordeaux, ISPED, France, Centre INSERM U1219, Bordeaux, France; 5 Institut de Cardiologie d'Abidjan, Côte d'Ivoire; 6 Centre Intégré de Recherche Bioclinique d'Abidjan, Côte d'Ivoire; 7 Service de Cardiologie, Hôpital Saint Antoine Paris, France, INSERM 938 Université Pierre et Curie, Paris, France

**Keywords:** VIH, adultes, pathologie vasculaire, anévrysme, dissection aortique, syndrome de restauration immunitaire, tuberculose, antirétroviral, Afrique, HIV, adults, vascular pathology, aneurysm, aortic dissection, immune reconstitution inflammatory syndrome

## Abstract

Un homme de 35 ans, VIH-1, sans antécédents médicaux et chirurgicaux particuliers, a été hospitalisé à Abidjan, Côte d'Ivoire, dans un contexte fébrile, toux, dyspnée, douleurs thoraciques et à la radiographie pulmonaire, un déroulement de la crosse de l'aorte une semaine après avoir débuté les antirétroviraux (ARV). Les scanners angiothoraciques réalisés ont mis en évidence une ectasie aortique globale étendue avec thrombus mural. Une échocardiographie transoesophagienne conclut à une dissection aortique, type A de Stanford. Le diagnostic de tuberculose a été confirmé par l'isolation en culture de *Mycobacterium Tuberculosis*. Huit ans après, le patient est encore vivant, sans intervention chirurgicale et se plaint de douleurs thoraciques intermittentes. Sa pression artérielle est stable et a une insuffisance rénale modérée. Nous rapportons un cas rare de dissection aortique anévrismale chez un adulte infecté par le VIH-1 dans le cadre d'un syndrome de reconstitution immune avec tuberculose pulmonaire.

## Abstract (en)

We here report the case of a 35-year old man with HIV-1 but with no previous medical-surgical history hospitalized in Abidjan, Côte d'Ivoire, due to fever, cough, dyspnea, chest pain and unfolding of the aortic arch observed on chest x-ray a week after having started antiretroviral therapy (ART). CT angiography of the thoracic aorta showed overall, extended aortic ectasia with mural thrombus. Transesophageal echocardiography objectified type A ascending aortic dissection (Stanford classification). The diagnosis of tuberculosis was confirmed based on Mycobacterium tuberculosis culture isolation. Eight years after, the patient was still alive without surgical treatment and complained of intermittent chest pain. Blood pressure was stable with moderate renal failure. We here report a rare case of aortic aneurism dissection in an adult patient with tuberculosis infected with HIV-1 during immune reconstitution inflammatory syndrome.

## Introduction

Les pathologies cardio-vasculaires sont de plus en plus fréquentes chez les sujets infectés par le VIH et sont causes d'aggravation de la morbidité et de la mortalité [1]. On estime que plus de 10% des personnes vivant avec le VIH pourraient présenter une pathologie cardio-vasculaire au cours de leur vie en raison de l'exposition aux antirétroviraux (par le biais de désordres métaboliques et dyslipidémiques), de l'action directe du virus (contrôlé ou non) par le rôle de l'inflammation chronique et de la réactivation immune [2, 3] ainsi que de l'augmentation de la survie des patients. Parmi les atteintes vasculaires pouvant être également infectieuses, se trouvent les anévrysmes mycotiques [4]. Ces anévrismes infectieux représentent une pathologie rare, mais potentiellement grave, siégeant préférentiellement au niveau de l'aorte et exposant au risque de sepsis sévère et de dissection aortique. En dehors des étiologies bactériennes connus, les mycobactéries et les agents fongiques (Candida, Aspergillus, Cryptococcus), des causes très rares d'anévrysmes mycotiques surviennent sur terrains immunodéprimés. Ce type de pathologies liées au VIH a été rarement décrit en Afrique sub-Saharienne. Nous décrivons ici, un cas clinique de dissection aortique chronique de type A avec anévrysmes artériels multiples détectée dans le cadre d'une tuberculose pulmonaire chez un adulte infecté par le VIH venant de débuter un traitement antirétroviral (ARV) et ayant survécu à ce jour sans une chirurgie réparatrice.

## Patient et observation

**Figure 1:**
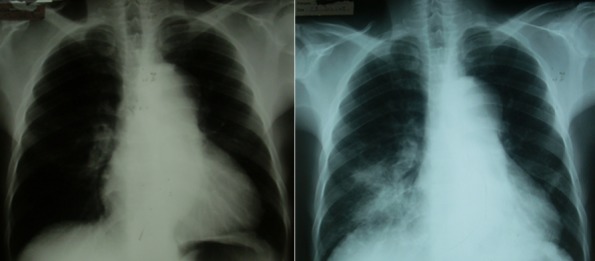
Radiographie de thorax initiale (gauche) et à J40 (droite)

Ce patient, africain, de nationalité ivoirienne, infecté par le VIH-1, a participé à l'essai Temprano ANRS. Cet essai évaluait les bénéfices et risques d'un traitement antirétroviral précoce (CD4 < 800/mm^3^) en comparaison avec un début selon les critères de l'OMS (avant Décembre 2009: CD4 < 200/mm^3^, ou stade 3 avec CD4 < 350, ou stade 4; à partir de Décembre 2009: CD4 < 350/mm^3^ ou stade 3-4 de l'OMS, à partir de Juin 2013: CD4 < 500/m^3^). A l'inclusion dans l'essai, le 19 Juin 2009, il a 35 ans, est au stade clinique 1 de l'OMS, il mesure 1,65 m et pèse 56 kg (indice de masse corporelle à 20,6 kg/m^2^). Il a une pression artérielle à 130/90 mmHg, un tabagisme estimé à 2 paquets/années et une consommation d'alcool estimée à 1-2 verres de bière par jour, mais pas de notion d'usage de drogues type cocaïne. La Numération Formule Sanguine (NFS), les transaminases sériques et la créatininémie sont normales. Le nombre de lymphocytes CD4 (True Count^®^ technique on FACScan^®^, Becton Dickinson) est à 289/mm^3^ (18,4%), et la charge virale plasmatique (PCR en temps réel Taq Man technology Abi Prism 7000, Applied Biosystems) à 4,42 log_10_copies/ml. La radiographie pulmonaire montre un déroulement de la crosse aortique, sans anomalie parenchymateuse pulmonaire (Figure 1).

**Figure 2:**
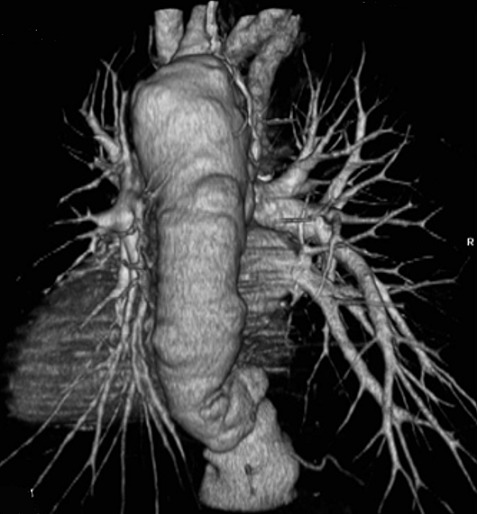
Angiographie thoracique (N°1), 16 Juillet 2009

Ce patient débute un traitement ARV par ténofovir-emtricitabine-efavirenz le jour de l'inclusion dans l'essai (J0). A J7, il présente une toux avec des expectorations dans un contexte non fébrile. Devant les images radiologiques à type de cardiomégalie, et l'apparition d'une dyspnée d'effort, à J19 une échographie cardiaque est réalisée, mettant en évidence une dilatation modérée des cavités cardiaques droites en télédiastole (VD: 33 mm, avec un rapport VD/VG > 0,6), des cavités gauches de taille normale, des parois non épaissies, et une hypertension artérielle pulmonaire modérée (pression artérielle systolique pulmonaire à 50 mmHg estimée sur le flux d´insuffisance tricuspide) et la fraction d´éjection ventriculaire gauche était à 55%. A J26, devant l'aggravation des symptômes (fièvre, douleurs thoraciques, palpitations, augmentation de la dyspnée d'effort), le patient est hospitalisé. Un angioscanner thoracique montre alors une ectasie aortique globale étendue (aorte thoracique mesurant 51mm de diamètre transversal) avec thrombus mural (Figure 2). Entre J26 et J40 apparait une fièvre à 40°C et le patient a perdu 7 kilos. A J40, la radio de thorax montre un foyer parenchymateux de la base droite (Figure 1). Un traitement antibiotique parentéral par Ceftriaxone (2g/jour) pendant 15 jours et Gentaline (150 mg/jour) pendant 5 jours est administré. A J48, le diagnostic de tuberculose est confirmé par la présence de Bacille Acido-Alcoolo-Resistant (BAAR) à l'examen direct d'une aspiration gastrique (qui sera positive en culture pour *Mycobacterium tuberculosis*), et un traitement antituberculeux standard (Rifampicine, Isoniazide, Pirazinamide, Ethambutol) est institué. Les sérologies Coxiella, Bartonella, borriellose, cytomégalovirus et VDRL sont négatives. Le TPHA est positif à 1/80. Les 5 hémocultures réalisées sont stériles. Les CD4 sont à 535/mm^3^ à J51 (+246 CD4 depuis le début des ARV).

### Evolution

**Figure 3:**
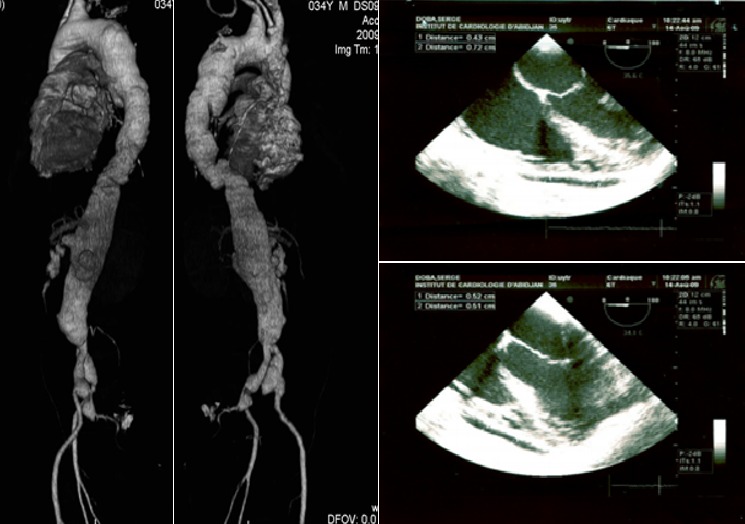
Angiographie thoraco-abdominale (N°2), 25 Août 2009

**Figure 4:**
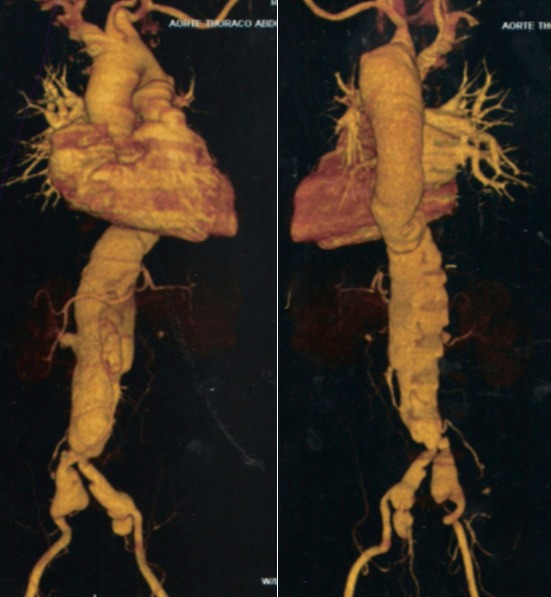
Angiographie thoraco-abdominale (N°3), 4 Février 2010

L'évolution sous traitement antituberculeux est favorable: apyrexie au bout de six jours, sortie d'hospitalisation, bonne observance au traitement, reprise de poids et disparition de la toux et de la dyspnée. Après six mois de traitement antituberculeux, le patient est déclaré guéri. Le reste de l'histoire concerne exclusivement l'aspect vasculaire. A partir de J48, une surveillance systématique est instituée et le bilan d'extension est complété. A J56, une échocardiographie transoesophagienne conclut à une dissection aortique, type A de Stanford. A J67, un angioscanner thoraco-abdominal montre des images thoraciques superposables à celles de l'examen de J26, alors qu'au niveau abdominal les images anévrismales s'étendent jusqu'aux artères rénales, à l'artère mésentérique supérieur et aux artères iliaques (Figure 3). A J90, le patient développe une hypertension artérielle à 160/100 mmHg et une insuffisance rénale, avec clairance de la créatinine à 33 ml/mn. L'hypertension artérielle nécessitera une trithérapie pour être contrôlée. Un angioscanner de contrôle réalisé à J230 montrera des images superposables aux précédents (Figure 4).

**Figure 5:**
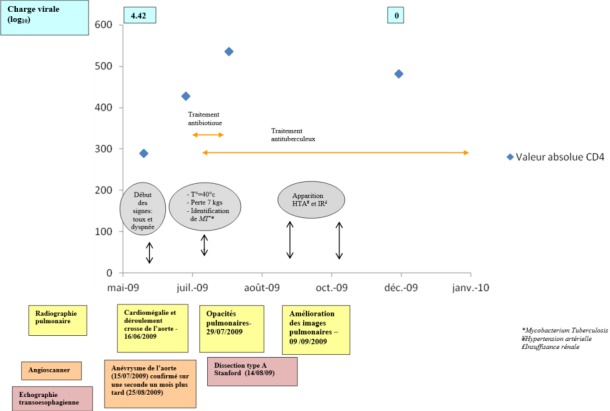
Évolution clinique, immuno-virologique du patient pendant la période aiguë

A ce jour, Mai 2017, 8 ans après la découverte de la pathologie, le patient est toujours vivant et suivi dans son centre de prise en charge pour le VIH. Il pèse 67 kg (indice de masse corporelle à 24,6 kg/m^2^). Il reçoit actuellement une trithérapie antirétrovirale à base de Abacavir-Lamivudine-Efavirenz. Le dernier bilan immuno-virologique est le suivant: CD4: 595-25%, charge virale < 40 copies/ml. Il se plaint de douleurs thoraciques intermittentes, parfois intenses, calmées par la trinitrine sublinguale. Sa tension artérielle est stable à 120/90 mmHg et sa clearance de la créatinine est à 53,3 ml/mn. La Figure 5 résume l'évolution du tableau, la séquence des symptômes, des examens réalisés et de l'évolution des CD4 et de la charge virale durant la phase aigüe.

## Discussion

Nous décrivons ici un cas clinique d'un sujet infecté par le VIH mis sous traitement antirétroviral et chez qui a été mise en évidence une dissection aortique anévrismale s'étendant sur toute la longueur de l'aorte, découverte dans un contexte de syndrome de restauration immune sous forme de tuberculose.

Différentes causes concernant la dissection aortique peuvent être évoquées. Une dissection aortique chronique pré-existante, de cause non infectieuse et non inflammatoire, et de découverte fortuite, est possible. La cause la plus fréquente de dissection aortique est l'hypertension artérielle (HTA). Dans notre cas, les paramètres échographiques cardiaques initiaux ne sont pas en faveur d'une HTA chronique pré-existante et l'HTA semble apparaitre secondairement chez notre patient. Cependant, les formes d'HTA masquée existent chez des personnes de faible indice de masse corporelle, ce qui pourrait être le cas chez notre patient. Dans la littérature, les autres causes anatomiques de dissection aortique sont les syndromes de Turner, de Noonan et de Marfan, des malformations congénitales telles qu'une coarctation de l'aorte, la persistance du canal artériel ou une bicuspidie de l'aorte, ou les traumatismes. Aucun élément du tableau de notre patient n'évoque une de ces causes. Une cause inflammatoire ou infectieuse est plus probable. Dans ce cas, deux questions se posent: le tableau est-il en rapport avec l'infection par le VIH lui-même, et peut-il avoir été provoqué ou démasqué par le contexte de restauration immune ?

Le VIH peut être à l'origine de pathologies artérielles de gros calibre par le biais d'une atteinte directe par le virus lui-même, de l'inflammation associée à la réplication virale, de mécanismes autoimmuns, ou de mécanismes infectieux par des pathogènes associés à l'origine d'anévrismes mycotiques [1, 5]. Dans ce dernier cas, l'étiologie bactérienne ou liée à un autre germe que le bacille tuberculeux doit être évoquée. Cependant, elle est peu probable ici au vue de l'évolution, le traitement antibiotique court reçu pendant la phase initiale ne suffisant pas à expliquer la stabilisation ultérieure: les deux traitements reçus par notre patient sont le traitement antituberculeux et le traitement antirétroviral; si on admet l'hypothèse d'une pathologie aortique évolutive, sa stabilisation ultérieure sous traitement rend donc probable qu'elle ait été liée au VIH lui-même, directement ou indirectement par le biais de l'inflammation, soit à la tuberculose [6].

Les atteintes des gros vaisseaux d'origine tuberculeuse décrits dans la littérature sont souvent par contamination adjacente, à l'origine de foyers extrapulmonaires, péricardiques, abcès para-vertébraux, spondylodiscite, ou empyème [7, 8]. Même si le tableau était essentiellement pulmonaire, il n'est pas impossible qu'un foyer de ce type soit passé inaperçu même si au niveau thoracique l'angioscanner ne notait ni anomalies parenchymateuses, ni épanchement pleural, ni adénopathies médiastinales. Mais on peut également évoquer une contamination endovasculaire avec aortite tuberculeuse, favorisée par un contexte de restauration immunitaire. Notre patient a en effet développé les premiers signes de tuberculose une semaine après le début des ARV et dans un contexte de traitement ARV efficace avec remontée rapide des CD4, faisant évoquer un syndrome de restauration immunitaire [9]. La survie des dissections aortiques est estimée entre 52 et 94% à un an et 45 et 88% à 5 ans. Presque huit ans après le début des troubles, notre patient est toujours vivant; sans intervention chirurgicale: la dissection touchant toute l'aorte et vu le caractère très volumineux de l'anévrisme [3, 10]. Un patient chinois, lui, est décédé 9 mois après le diagnostic de sa dissection aortique [3].

## Conclusion

Au total cette observation a deux intérêts: elle relance l'attention sur la pathologie vasculaire chez les patients infectés par le VIH, dont la fréquence est probablement sous-estimée; et elle attire l'attention sur les formes éventuellement très diverses et atypiques que peuvent prendre la tuberculose, le syndrome de restauration immunitaire et les atteintes des gros vaisseaux liées au VIH.

## Conflits d'intérêts

Les auteurs ne déclarent aucun conflit d'intérêts.

## Remerciements

Les auteurs ne déclarent aucun conflit d'intérêts.
